# A role for the medial temporal lobe subsystem in guiding prosociality: the effect of episodic processes on willingness to help others

**DOI:** 10.1093/scan/nsz014

**Published:** 2019-02-26

**Authors:** Brendan Gaesser, Josh Hirschfeld-Kroen, Emily A Wasserman, Mary Horn, Liane Young

**Affiliations:** 1 Department of Psychology, University at Albany, State University of New York, Albany, NY, USA; 2 Department of Psychology, Boston College, Chestnut Hill, MA, USA

**Keywords:** prosocial, episodic simulation, memory, medial temporal lobes, moral cognition

## Abstract

Why are we willing to help others? Recent behavioral work on episodic processes (i.e. the ability to represent an event that is specific in time and place) suggests that imagining and remembering scenes of helping a person in need increases intentions to help. Here, we provide insight into the cognitive and neural mechanisms that enhance prosocial intentions via episodic simulation and memory. In Experiment 1, we scanned participants using functional neuroimaging as they imagined and remembered helping episodes, and completed non-episodic control conditions accounting for exposure to the story of need and conceptual priming of helping. Analyses revealed that activity in the medial temporal lobe (MTL) subsystem, as well as the right temporoparietal junction (RTPJ) predicted the effect of conditions on the strength of prosocial intentions. In Experiment 2, we used transcranial magnetic stimulation to disrupt activity in the RTPJ, and better isolate the contribution of MTL subsystem to prosocial intentions. The effect of conditions on willingness to help remained even when activity in the RTPJ was disrupted, suggesting that activity in the MTL subsystem may primarily support this prosocial effect. It seems our willingness to help may be guided, in part, by how easily we can construct imagined and remembered helping episodes.

## Introduction

Humans often collaborate, coordinate and help each other in times of need. Research in social neuroscience and psychology has focused on investigating how the processes underlying our perceptions of people in need, their mental states and our subsequent emotional reactions contribute to prosociality (Coke *et al*., 1978; Decety, 2005; Singer and Lamm, 2009; Batson, 2011; Rameson *et al*., 2012; Waytz *et al*., 2012; Zaki and Ochsner, 2012; Marsh, 2016). Great progress has been made in revealing how neural mechanisms associated with various social-cognitive processes, including theory of mind/mentalizing (Masten *et al*., 2011), representing individual victims (Genevsky *et al*., 2013), affect-sharing (Singer and Lamm, 2009; Hein *et al*., 2010) and positive empathy (Morelli *et al*., 2015), can inform decisions to help others. But, helping involves more than representing and reacting to a person in isolation: it involves a specific event unfolding in time and place, in which the person is embedded. While person-centric accounts of prosociality have yielded key insights, they do not address the potential importance of how the helping event itself is represented. Thus, the current work aims to investigate the following questions: Does it matter not just how we represent the person in need, but how we represent the episodic details of helping? Can neural systems that support episodic simulation (imagining hypothetical and future events) and episodic memory (remembering past events) contribute to our willingness to help others?

Episodic representation involves consciously experiencing an unfolding sequence of details (e.g. people, objects) in a specific place as an event or scene (Schacter *et al*., 2008). Whereas episodic memory is retrospective, episodic simulation is less anchored to a temporal direction, including imagining possible future events (Atance and O’Neill, 2001; Seligman *et al*., 2013; Szpunar *et al*., 2014), atemporal fictitious events (Hassabis *et al*., 2007; Summerfield *et al*., 2009, 2010) and counter-factual events (De Brigard *et al*., 2013). A large body of neuroimaging work (Addis *et al*., 2007; Szpunar *et al*., 2007; see Benoit & Schacter, 2015 for meta-analysis) and patient studies (Tulving, 1985; Klein *et al*., 2002; Hassabis *et al*., 2007; D’argembeau *et al*., 2008; Addis *et al*., 2009; Berryhill *et al*., 2010; de Vito *et al*., 2012; Race *et al*., 2011) has established that episodic simulation and memory recruit many of the same brain regions, including the medial temporal lobes (MTLs), medial prefrontal cortex (mPFC), posterior cingulate cortex (PCC), lateral temporal cortex and lateral parietal cortex, commonly referred to as the default network (Raichle et al., 2001; Buckner and Carroll, 2007; Spreng *et al.*, 2009). This network is comprised of dissociable subsystems (i.e. the core, dMPFC and MTL subsystems) that show different patterns of connectivity at rest and support different component processes (Andrews-Hanna *et al*., 2010; Andrews-Hanna *et al*., 2014). The core subsystem is extensively connected to all other regions across the default network. The dMPFC subsystem, comprised of the dMPFC, lateral temporal cortex and temporal pole, is recruited for semantic knowledge, mental state content and narrative processing. Most pertinent to the present study is the MTL subsystem, comprised of the hippocampus, parahippocampus, retrosplenial cortex, posterior inferior parietal lobule and, to some extent, the ventral medial prefrontal cortex (vMPFC), is critical for imagining and remembering events. The MTL subsystem is particularly engaged when participants generate episodes specific in time and place and is sensitive to scene construction (i.e. the need to embed an event within a spatial context; Andrews-Hanna *et al*., 2010; Addis *et al*., 2011; Madore *et al*., 2016; Tamir *et al*., 2016; Palombo *et al*. 2018).

While cognitive neuroscientists have learned a great deal about the brain systems supporting episodic processes, little is known about their potential contribution to social cognition, broadly and prosociality, more specifically. Emerging behavioral evidence finds that imagining future events and remembering past events can facilitate prosocial intentions to help a person in need: participants were more willing to help a person in need after imagining helping in that situation or remembering helping in a related situation (Gaesser *et al*., 2015; Gaesser and Schacter, 2014; Gaesser *et al*., 2017a, 2017b). A shared cognitive mechanism has been suggested to underlie the similar effects of both imagining and remembering on prosocial intentions. Initial evidence has come from behavioral measures suggesting that, as the imagined and remembered helping scene becomes more vividly represented, the accessibility of the helping event and subjective plausibility that one will help in that situation increases. Relatedly, amnesic patients with damage to the MTL, often characterized by parallel deficits in imagining future events and remembering past events, have been shown to exhibit prosocial deficits in some cases (Beadle *et al*., 2013).

While the MTL subsystem may directly influence prosocial intentions by heightening access and the subjective plausibility of a helping episode, another possibility is that the MTL subsystem guides prosocial intentions by interacting with other person-focused mechanisms previously shown to impact prosociality. Indeed, we have recently found behavioral evidence that an effect of episodic simulation on prosocial intentions and decision-making may be partially attributed to an increase in considering the mental states of the person in need (i.e. theory of mind akin to mentalizing, perspective-taking) for the person in need (Gaesser *et al*., 2018). For example, imagining or remembering a helping episode may recruit theory of mind, making it easier to consider the thoughts and feelings of the person in need embedded within the imagined or remembered episode. On this account, imagining and remembering helping a person in need may elicit enhanced activity in the network of regions that support theory of mind (akin to mentalizing or perspective-taking), such as the right temporal parietal junction and dorsal mPFC (Saxe and Wexler, 2005; Scholz *et al*., 2009; Young *et al*., 2010; Zaki and Ochsner, 2012; Hill *et al*., 2017).

Here, we investigated the neural basis of the prosocial effect of episodic processes using functional magnetic resonance imaging (fMRI; Experiment 1) and transcranial magnetic stimulation (TMS; Experiment 2) in order to examine whether neural regions engaged during episodic simulation and episodic memory can guide a willingness to help others. Across both experiments we used a theory of mind functional localizer to independently define each participant’s theory of mind network to interrogate (Experiment 1) and disrupt (Experiment 2) activity in this network. Because no such functional localizer exists for episodic simulation and episodic memory, we instead targeted the MTL subsystem coordinates independently defined in previous work using functional connectivity (Andrews-Hanna *et al*., 2010) and associated with imagining and remembering specific episodes or scenes (Andrews-Hanna *et al*., 2010; Addis *et al*., 2011; Madore *et al*., 2016; Tamir *et al*., 2016; Palombo *et al*., 2018).

Given that the MTL subsystem of the default network supports constructing mental scenes of episodic experiences, one possibility is that activity within the MTL subsystem would inform a willingness to help. Another possibility is that generating episodic experiences recruits theory of mind, and it is instead activity within the theory of mind network that informs a willingness to help. Thus, the present study sought to investigate the neural basis of the prosocial effect of episodic processes and gain insight into the underlying mechanism.

## Experiment 1

### Participants and procedures

Twenty-two, right-handed healthy adults with normal or corrected to normal vision were recruited from a community sample to participate in Experiment 1. All participants provided written informed consent in accordance with the Boston College Institutional Review Board. Participants were paid $25 per h as compensation. Participants were scanned on a 3 T Siemens Magnetom Tim Trio MRI scanner with a 32-channel phase-array whole-head coil (at the Center for Brain Science at Harvard University) using 49 slices covering the whole brain with 3 × 3 mm in-plane resolution collected perpendicular to the long axis of the hippocampus. Standard echoplanar imaging procedures were used [repetition time (TR) = 2.5 s, echo time (TE) = 25 ms, flip angle 85°] with integrated parallel imaging techniques (iPaT = 2). Blood Oxygen Level-Dependent (BOLD) scans were motion-corrected in real-time using a Prospective Acquisition CorrEction protocol. Before BOLD scans began, two rapid images were collected to create a pre-scan map of displacement in the MR signal (voxel distortion), which then applied distortion correction to the subsequent BOLD scans to increase SNR (Holland *et al*., 2010). The scanning parameters and procedures were informed by the head of MR Physics at the center in order to optimize signal detection with the MTL, while still enabling whole-brain coverage.

Participants who failed to complete the study (e.g. one subject felt nauseous during scanning and did not complete the study) or provide a fully usable data set (e.g. we were unable to localize the theory of mind network in three subjects) could not be used for data analysis. Eighteen participants (age 18–34 years, *M* = 23.56 years, *s.d.* = 4.68, six males) provided full data sets that were then used for analysis. A power analysis of the effect size (*d* = 1.32) for the primary contrast of interest in the most relevant prior work (i.e. the difference in willingness to help for episodic *vs* control conditions, *n* = 15; Gaesser and Schacter, 2014) indicates that running 18 participants would allow for adequate power to detect behavioral differences across conditions (power > 0.80). At the end of the study, participants were compensated and thanked for their participation. Behavioral data sets for Experiments 1 and 2 are available on the Open Science Framework at https://osf.io/9k4n7/, and the fMRI data set for Experiment 1 is available on Open Neuro at https://openneuro.org/datasets/ds001439.

Participants were informed of the purpose of the study in broad terms: to examine reactions to stories from online media. Participants were scanned while reading and responding to a series of 40 stories depicting people in need (e.g. a passenger is harassed on the subway, see Supplementary Material for stories). After reading each story depicting a person in need for 10 s, participants had 60 s to complete one ‘episodic’ task (Imagine Helping, Remember Helping) or one ‘control’ task (No Helping, Conceptual Helping). Stories were randomly assigned across conditions. Participants read 10 stories per condition. Information about gender and race (variables shown to influence empathic responding) were absent. Stories were then removed from the screen and replaced with prompts to complete one of the experimental tasks in a randomized order: (i) consider the writing style and media source of the story (No Helping-control condition), (ii) estimate and visualize comments that could be posted on the website describing how the person could be helped (Conceptual Helping-control condition), (iii) imagine a future event of helping the person in need (Imagine Helping-episodic condition) or (iv) remember a past event of helping someone in need similar to the current media story (Remember Helping-episodic condition). The No Helping condition, modeled after control conditions commonly used in empathy research (e.g. Coke *et al*., 1978), aims to control for exposure to the person-in-need’s plight while preventing the participant from actively thinking about helping the person. The Conceptual Helping condition (akin to the Estimate Helping condition in Gaesser and Schacter, 2014) was designed to recruit semantic retrieval, social cognition and the conceptual priming of helping responses (Macrae and Johnston, 1998; Nelson and Norton, 2005). Moreover, by having subjects ‘visualize’ the media website and corresponding comments, the Conceptual Helping condition controls for generating basic visual imagery (Kosslyn *et al*., 2001). In contrast, the Imagine Helping and Remember Helping conditions required generating an episode that is specific in time and place. Indeed, these conditions were directly informed by previous neuroimaging and behavioral research using similar instructions and protocol to successfully prompt subjects to generate events set in a specific time and place (e.g. Addis *et al*., 2007, 2011; Gaesser and Schacter, 2014).

Participants had 60 s to complete each experimental task, after which they used a button box to rate ‘task difficulty’: how difficult was it to generate a response? (1 = not very difficult to 7 = very difficult). This rating served as general indicator of subject attentiveness on a trial-by-trial basis during scanning. Each trial (story + experimental task + rating) was randomly interleaved with 3, 6 or 9 s of fixation, allowing for an event-related analysis by establishing temporal jitter in the experimental design. Experimental trials consisted of eight functional runs lasting 7 min 44.4 s. At the end of the same scan session, two runs of a theory of mind functional localizer were collected to define each participant’s theory of mind network (Saxe and Kanwisher, 2003).

In a post-scan behavioral session, participants re-read each person-in-need story and provided brief descriptions of the experiences they generated in the scanner. Trials on which participants failed to generate an event were not included in analyses consistent with past work (Gaesser and Schacter, 2014; Gaesser *et al*., 2015; Gaesser *et al*., 2017a, 2017b; see Supplementary Behavioral Results for related data and discussion on episodic flexibility). In addition to brief descriptions, participants rated their ‘willingness to help’ (e.g. How likely would you be to help in this situation?; 1 not at all–7 very willing) for each story. Participants rated their ‘scene imagery’ of the imagined and remembered events, for scene coherence (e.g. The imagined scene in your mind was?; 1 vague–7 clear and coherent) and scene detail (e.g. The imagined scene in your mind was?; 1 simple–7 detailed). Participants also rated their imagery of the media website that comments could be posted on describing how the person could be helped for the Conceptual Helping-control condition (i.e. The media website in your mind was?; 1 vague– clear and coherent; The media website in your mind was?; 1 simple–7 detailed). Participants rated the degree to which they engaged in perspective-taking (i.e. theory of mind, mentalizing, cognitive empathy; e.g. when imagining helping did you consider the person’s thoughts and feelings? 1 = not at all–7 = strongly considered). Descriptions were collected at the end of the study instead of completed online (i.e. directly after each scenario) to maximize time in the scanner during which subjects completed episodic and control tasks. Previous research and pilot testing found that subjects are able to reliably reflect back on similar experiences generated during the experiment in the scanner (Addis *et al*., 2007; Martin *et al*., 2011). Most pertinent for addressing concerns of retrospective *vs* online ratings are behavioral studies that did collect ratings online (Gaesser *et al*., 2015, 2018), and found that generating helping episodes had a similar effect on online ratings of willingness to help.

### fMRI analyses

Functional images were preprocessed and analyzed using SPM12 (http://www.fil.ion.ucl.ac.uk/spm). Each subject’s functional images were first preprocessed to correct for slice-timing differences. Each subject’s T1-weighted structural scan was coregistered and normalized to Montreal Neurological Institute (MNI) brain space. These parameters were then used to normalize each subject’s functional images into MNI space, which was then smoothed using a Gaussian kernel (full width half maximum 6 mm) and high-pass filtered for analysis. Preprocessed images were analyzed using a slow event-related design with events modeled using a boxcar regressor to estimate the hemodynamic response for each condition. An event was defined as the helping task (Imagine Helping-episodic, Remember Helping-episodic, Conceptual Helping-control or No Helping-control) for an individual trial, and the event onset was defined by the onset of text on screen. Each helping task was preceded by a story adapted from online media, holding constant exposure to a person in need across trials while varying thoughts directed at helping that person. The timing of the story and subsequent helping task was the same for every trial, so independent parameter estimates could not be created for each component. Helping tasks were instead isolated by accounting for hemodynamic lag. Covariates of no interest (session mean and linear trend) were also included in the model. Preprocessed data were analyzed with whole-brain general linear modeling and tailored regions of interest (ROIs) analyses. Analyses were conducted individually for each participant, and contrast images were then entered into a second-level analysis, treating participants as a random effect.

In the same scan session as the main experiment we ran a theory of mind functional localizer (Saxe and Kanwisher, 2003; Dodell-Feder *et al*., 2011). Based on this independent data set, we defined individually tailored functional ROIs for ToM (i.e. brain regions supporting the representation of mental states). ROIs were defined for each subject based on a whole-brain analysis of the theory of mind localizer. We defined ROIs as all voxels within a 9 mm radius of the peak voxel that survived threshold in the contrast of mental state (i.e. belief) stories over control (photo) stories (*P* < 0.001, uncorrected, *k* > 15, setting an extent threshold using non-parametric permutation testing). Specifically, we computed this threshold via 1,000 iterations of a Monte Carlo simulation (Slotnick *et al*., 2003). Within each ROI, we averaged across voxels to extract a single time course of BOLD response. A baseline value was calculated as the average ROI response across all inter-stimulus time points.

Given that no functional localizer exists for episodic simulation and episodic memory, we instead targeted the MTL subsystem coordinates independently defined in previous work using functional connectivity (Andrews-Hanna *et al*., 2010). Specifically, we conducted ROIs analyses using 9 mm spheres centered on the regions comprising the MTL subsystem, including the parahippocampus, hippocampus, retrosplenial cortex, posterior inferior parietal lobule, and vMPFC (Andrews-Hanna *et al*., 2010; Supplementary Table 1).

All behavioral analyses were conducted in R v.3.2.4, involving both base and custom software as well as the packages lme4 (Bates *et al*., 2014), afex (Singmann *et al*., 2016) and lsmeans (Lenth, 2016). See Supplemental Materials for reporting of whole-brain contrasts during the episodic helping task.

## Results

### Behavioral results

#### Effect of episodic processes on willingness to help

To assess the effect of episodic processes on willingness to help, scene imagery and perspective taking while controlling for variability in mean responses by subject and by scenario, linear mixed models were fit with a fixed effect for condition and random-effects terms for subject and scenario. Models that included a continuous independent variable also contained random-slopes terms, to control for differences in the independent-dependent variable relationship across subjects or scenarios. To estimate *P*-values for fixed effects, the Kendall–Rogers approximation for degrees of freedom was implemented with afex::mixed. Pairwise *post hoc* tests were Tukey-corrected for multiple comparisons, as implemented in lsmeans.

**Fig. 1:**
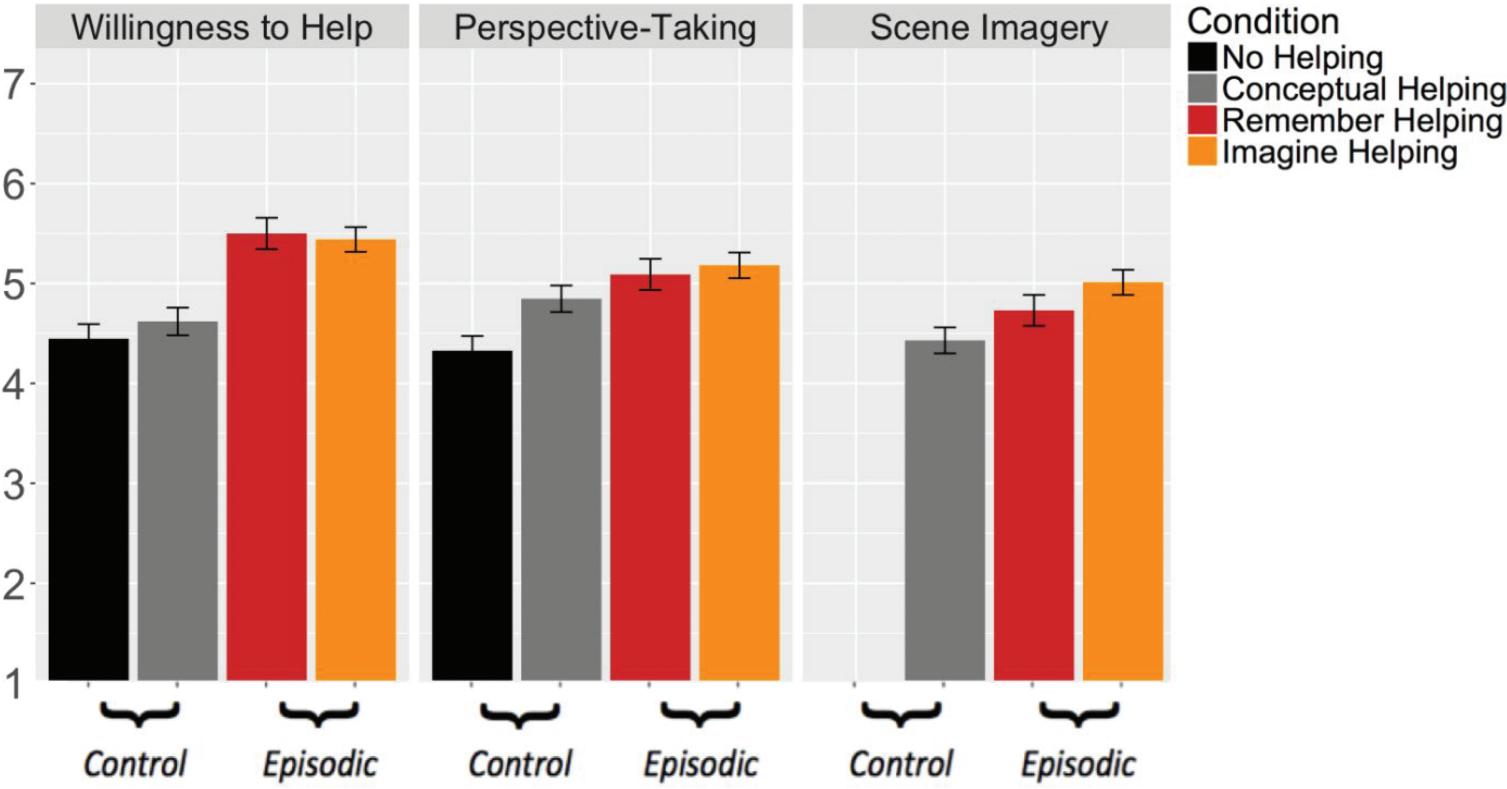
Mean willingness to help, perspective-taking and scene imagery by condition. Willingness to help was significantly higher for ‘episodic’ compared to ‘control’ conditions. ‘episodic’ conditions were matched on the degree of willingness to help, perspective taking and scene imagery evoked by the helping scenario. Error bars indicate standard error of the mean.

Willingness to help differed significantly across ‘episodic’ and ‘control’ conditions (*F*(38.11) = 46.88, *P* < 0.001, *d* = 0.51), due to higher willingness to help in ‘episodic’ (*M* = 5.47) than in ‘control*’* conditions (*M* = 4.53) conditions (Figure 1). In pairwise tests, the ‘episodic’ conditions Imagine Helping (*M* = 5.44) and Remember Helping (*M* = 5.50) increased willingness to help to a similar extent (*t*(41.34) = 0.61, *P* = 0.93, *d =* 0.03)*.*

#### Scene imagery and perspective-taking

Scene imagery and perspective-taking varied significantly by condition [SI: *F*(26.06) = 8.26*, P =* 0.01, *d =* 0.27; PT: *F*(38.12) = 22.67*, P <* 0.001, *d* = 0.30] such that scene imagery and perspective-taking were higher for ‘episodic’ conditions (SI: *M* = 4.89; PT: *M* = 5.14) compared to ‘control’ conditions (SI: *M* = 4.43; PT: *M* = 4.59; Figure 1)*.* Scene imagery and perspective-taking were similarly recruited in the Imagine Helping and Remember Helping conditions [SI: *t*(30.43) = 1.01, *P* = 0.60, *d =* 0.16; PT: *t*(43.76) = 0.07, *P* = 0.99, *d =* 0.05]. Thus, our ‘episodic’ conditions were matched on the degree of willingness to help and phenomenology of the helping scenario they evoked.

### fMRI results

#### Definition of functional ROIs

Whole-brain results for the theory of mind localizer (thresholded at *P* < 0.001, *k* > 15, in line with standard functional ROI methods) yielded a network of brain regions (Supplementary Table 3) that have been reliably observed in previous work (Saxe and Wexler, 2005; Scholz *et al*., 2009; Young *et al*., 2010; Schurz *et al*., 2014): right and left TPJ, right and left superior temporal sulcus (STS), precuneus and medial prefrontal cortex (MPFC; see Methods for the MTL subsystem-defined ROIs).

#### Linear mixed models for brain–behavior relationships

Separate mixed-effects models for the relationship between each ROI mean BOLD signal, drawn from itemwise beta images and willingness to help were conducted in R using the lme4, afex and multcomp packages. Each model included the fixed-effects terms for condition and mean BOLD response, and additionally included random intercepts and slopes for subject and item to ensure that fixed effects did not reflect variance due to these factors. For models that failed to converge due to overfitting, data were remodeled with random intercepts only. Fixed effect statistics and results of model comparisons for models on willingness to help are reported in Table 1. Following model fitting, significance of the mean BOLD term was assessed via likelihood-based model comparison with a version of the model in which neural effects were removed. Significance of each fixed effect was estimated using the Kenward–Roger approximation for degrees of freedom (Judd *et al*., 2012).

**Table 1:** Linear mixed-effects models for effects of ROI BOLD signal on willingness to help

Predictor BOLD	Fixed effect of interest	*F*	*df*	*p*		Model comparison *X^2^*	Model comparison *p*	*N* localized
MTL subsystem								
Parahippocampus R.	Condition	49.31	37.86	< 0.001	***	8.69	0.2	
	BOLD predictor	0.87	8.7	0.38				
	Condition x BOLD	6.84	30.6	0.01	**			
Parahippocampus L.	Condition	47.03	39.03	< 0.001	***	11.06	0.09	
	BOLD predictor	1.62	12.45	0.23				
	Condition x BOLD	5.4	34.75	0.03	*			
Hippocampus R.*	Condition	54.9	41.52	< 0.001	***	11.12	0.003	
	BOLD predictor	0.32	647.21	0.57				
	Condition x BOLD	10.99	645.08	0.001	***			
Hippocampus L.	Condition	52.85	38.54	< 0.001	***	15.09	0.02	
	BOLD predictor	0.28	21.48	0.6				
	Condition x BOLD	6.56	37.51	0.01	*			
Retrosplenial R.*	Condition	40.37	44.28	< 0.001	***	2.55	0.29	
	BOLD predictor	1.71	655.11	0.19				
	Condition x BOLD	0.96	646.4	0.33				
Retrosplenial L.*	Condition	44.36	40.37	< 0.001	***	2.56	0.28	
	BOLD predictor	2.26	651.54	0.13				
	Condition x BOLD	0.37	647.91	0.54				
VMPFC	Condition	46.45	38.93	< 0.001	***	6.02	0.42	
	BOLD predictor	0.54	14.95	0.47				
	Condition x BOLD	3.4	41.37	0.07	†			
Posterior IPL R.	Condition	47.02	38.15	< 0.001	***	9.46	0.15	
	BOLD predictor	0.15	13.08	0.71				
	Condition x BOLD	4.63	35.81	0.04	*			
								
Posterior IPL L.	Condition	45.97	38.44	< 0.001	***	7.63	0.27	
	BOLD predictor	1.73	13.11	0.21				
	Condition x BOLD	1.9	27.96	0.18				
								
ToM network								
RTPJ*	Condition	45.96	39.55	< 0.001	***	5.04	0.08	18
	BOLD predictor	2.66	642.05	0.1				
	Condition x BOLD	5.02	648.43	0.03	*			
DMPFC	Condition	27.89	39.93	< 0.001	***	4.43	0.62	12
	BOLD predictor	0.51	19.61	0.48				
	Condition x BOLD	0.02	34.78	0.89				
MPFC	Condition	38	40.02	< 0.001	***	3.47	0.75	14
	BOLD predictor	0.02	16.54	0.88				
	Condition x BOLD	1.52	33.34	0.23				
STS R.	Condition	50.96	38.52	< 0.001	***	1.46	0.96	15
	BOLD predictor	0.83	16.67	0.38				
	Condition x BOLD	0.79	32.48	0.38				
STS L.	Condition	38.6	37.36	< 0.001	***	1.57	0.95	9
	BOLD predictor	0	10.74	0.96				
	Condition x BOLD	0.49	27.44	0.49				
LTPJ	Condition	42.11	38.1	< 0.001	***	3.14	0.79	17
	BOLD predictor	2.35	16.4	0.14				
	Condition x BOLD	0.98	28.8	0.33				
PC*	Condition	36.71	39.54	< 0.001	***	1.96	0.37	16
	BOLD predictor	1.94	571.57	0.16				
	Condition x BOLD	0.68	578.34	0.41				

Note: df for all model comparisons = 6 (for models with random slopes) or 2 (for those without random slopes).

^*^model refit without random slopes to address overfitting (indicated by convergence failure)

The effect of condition (episodic *vs* control) on willingness to help was significant in all models (all *F* ≥ 27.81, all *P* < 0.001; Table 1), in accordance with the behavioral findings of enhanced willingness to help in ‘episodic’ conditions relative to ‘control’ conditions (see Behavioral Results). For regions in the theory of mind network, we largely did not observe differences in the relationship between BOLD activation and willingness to help between ‘episodic’ and ‘control’ conditions (Table 1). However, we did observe some evidence of condition by neural signal interaction in the right TPJ (RTPJ; *P* = 0.02), such that BOLD signal and willingness to help were more strongly negative in ‘episodic’ conditions than in ‘control’ conditions, suggesting RTPJ activity may have some role to play in the prosocial effect of episodic processes.

**Fig. 2:**
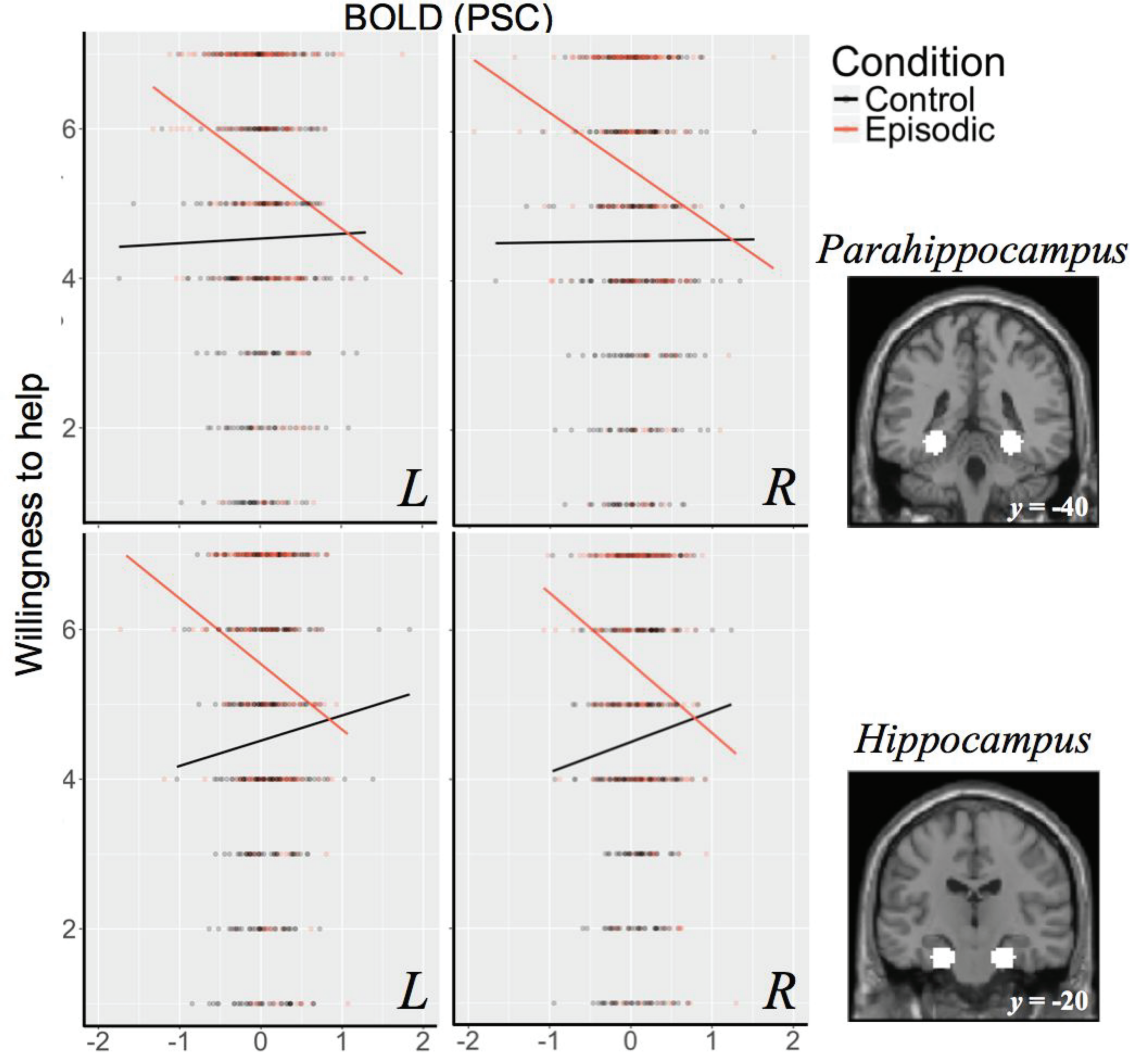
Relationship between BOLD percent signal change and willingness to help across episodic and control conditions in bilateral hippocampus (top) and hippocampus (bottom) with defined masks also shown. BOLD signal in the parahippocampus and hippocampus negatively predicted willingness to help during the episodic conditions but not during the control conditions (Andrews-Hanna *et al*., 2010).

Of the MTL subsystem regions, models including BOLD signal explained significantly more variance in willingness to help than the null model for left (*P* = 0.02) and right hippocampus (*P* = 0.003). The interaction of this neural signal with condition was significant in right parahippocampus (*P* = 0.01), left parahippocampus (*P* = 0.03), right hippocampus (*P* = 0.001) and left hippocampus (*P* = 0.01) and right posterior inferior parietal lobule (*P* = 0.04). Pairwise comparisons between all levels of the interaction factor, single-step corrected for multiple comparisons, indicated that the relationship between BOLD signal and willingness to help was more strongly negative in ‘episodic’ conditions than in ‘control’ conditions (*P*s < 0.05, see Figure 2).

#### The effect of difficulty on willingness to help

**Fig. 3:**
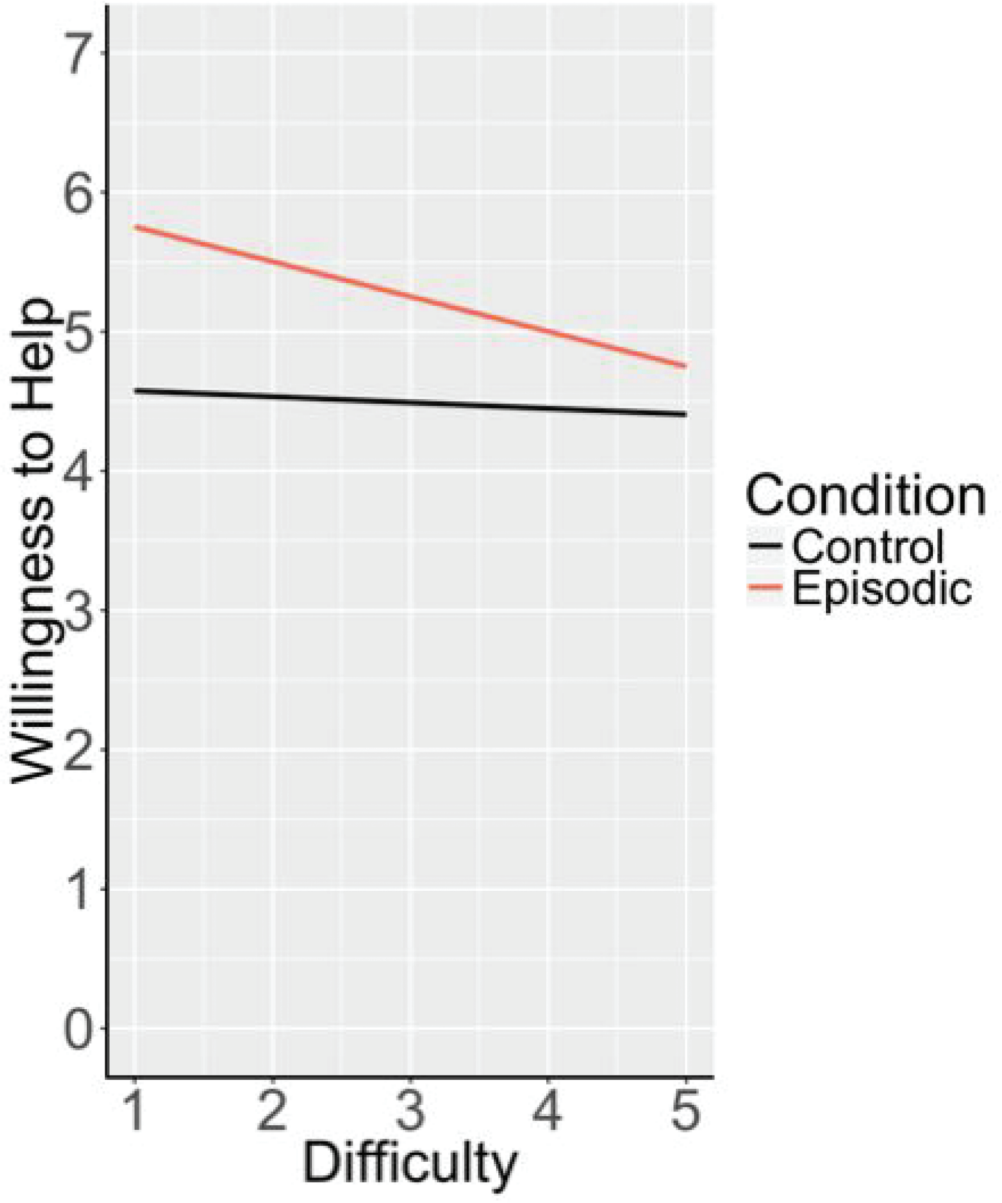
Relationship between difficulty and willingness to help by condition. Task difficulty significantly predicted willingness to help only for episodic, but not control conditions, suggesting that the more easily imagined and remembered helping episodes are constructed the more willing one is to help in that scenario.

The above results suggest an unpredicted inverse relationship between BOLD signal and willingness to help in the MTL subsystem regions during episodic helping. One plausible explanation involves differences in processing demands of episodic construction: the more easily disparate episodic details can be bound into an integrated event or scene, as reflected by reduced recruitment of activity in the MTL (Gaesser *et al*., 2013; van Mulukom *et al*., 2013; Szpunar *et al*., 2013), the greater the influence on a willingness to help. More simply put, the easier it is to construct and represent the imagined and remembered helping events, the more willing participants may be to help. To explore this possibility, we turned to the difficulty ratings of task completion provided by participants in the scanner to assess whether subjective difficulty of imagining and remembering helping events, as a proxy for ease of episodic construction, affected willingness to help. We found some evidence consistent with this interpretation. Specifically, the relationship between difficulty and willingness to help was significant and negative for episodic trials ‘only’ [*F*(253.32) = 4.51, *P* = 0.03, *β* = −0.20]. There was no relationship between difficulty and willingness to help in the control conditions, *F*(323.91) = 0.23, *P* = 0.63; *β* = 0.05. This pattern holds when miss trials are included in this analysis (i.e. potentially the most difficult trials in which participants failed to recall an episode of helping, see Supplemental Material); the relationship between difficulty and willingness to help remained significant and negative (*β* = −0.23) for episodic trials ‘only*’* [*F*(252.42) = 5.84, *P* = 0.02; Figure 3].

Adding difficulty ratings to the brain-behavior models for the MTL-subsystem ROIs where we found an interaction effect between BOLD and condition on willingness to help [i.e. bilateral hippocampus and parahippocampus and right posterior inferior parietal lobule (pIPL)] rendered the interaction effect null (*P*s ≥ .17) in all cases, supporting the interpretation that difficulty of constructing imagined and remembered helping episodes may play a role in the relationship between activity in these regions and willingness to help.

Overall, the data from Experiment 1 suggests that the MTL subsystem may contribute to prosociality: BOLD signal in the parahippocampus and hippocampus in particular predicted willingness to help during the episodic conditions but not during the control conditions. However, we also observed a similar pattern of results in a central node of the theory of mind network, the RTPJ. Follow-up connectivity analyses did not reveal evidence for functional coupling between activity in the MTL subsystem and the RTPJ (see Supplemental fMRI Results), suggesting dissociable recruitment of the MTL subsystem and the RTPJ. The similar patterns for these regions (i.e. condition by activity interaction), however, make it unclear whether the MTL subsystem or the RTPJ underlies the prosocial effect of episodic processes. Therefore, in Experiment 2, we used TMS to disrupt activity in the RTPJ, to better isolate the contribution of episodic processing, as supported by MTL subsystem, to prosocial intentions. We were specifically interested to see whether generating a helping episode would continue to increase willingness to help to a similar extent when activity in the RTPJ was disrupted compared to when it is intact. If the RTPJ is causally contributing to the prosocial effect of episodic processing, then willingness to help should decrease when activity in the RTPJ is disrupted. On the other hand, if the activity in the MTL subsystem primarily underlies the prosocial effect of episodic processing, then disrupting activity in the RTPJ should have no effect to little effect on willingness to help.

## Experiment 2

### Methods

#### Participants

For Experiment 2, a new set of participants were recruited from campus and surrounding community. All participants had normal, or corrected to normal vision, were native English-speakers, and were compensated $40 an hour for participation in the study. All participants signed an informed consent and TMS screening form prior to participating in both sessions of the experiment in accordance with the guidelines approved by the Institutional Review Board at Boston College. Each session lasted ~2 h. Twenty-three right-handed participants (ages 18–30) were run, three were excluded for failure to follow instructions (e.g. two subjects imagined helping on every trial, including control trials, and one was a no show for the second TMS session) and one was excluded due to machine malfunction (e.g. a blown fuse) that required the manufacturer to work on the machine before we could collect data for a second session. Nineteen total participants were included in the analyses (10 female, 7 male).

### Procedure: fMRI

Subjects were scanned on a 3 T Siemens Magnetom Tim Trio MRI at the Center for Brain Science at Harvard University using 26 4 mm-thick near-axial slices covering the whole brain. Standard echoplanar imaging procedures were used (TR = 2 s, TE = 40 ms, flip angle = 90°). Subjects participated in two runs of a theory of mind functional localizer that were used to define each participant’s theory of mind network in the same manner as in Experiment 1 and previous work (Saxe and Kanwisher, 2003; Dodell-Feder *et al*., 2011), yielding a network of brain regions, including the right and left TPJ, right and left STS, precuneus and mPFC. To identify the RTPJ for each participant, we defined the functional region as a sphere of contiguous voxels converging on the right lateralized junction of the temporal and parietal lobes in subjects’ native brain space that were significantly more active while the subject read mental state (i.e. belief) stories, as compared with control (i.e. physical object) stories (*P* < 0.001, *k* > 10, voxel-wise, uncorrected), consistent with Experiment 1 and previous work.

### Procedure: TMS

Offline TMS took place in McGuinn Hall at Boston College and closely followed the procedures of Young *et al*. (2010). Specifically, we used a Mag-Stim rapid2 stimulator and a commercially available, eight-shaped, 70 mm coil (MagStim Corporation). The intensity of stimulation was 60% of the stimulator’s maximum output for all subjects; the frequency was 1 Hz and the duration was 17 min. The coil was oriented in the anteroposterior axis with the handle pointing posteriorly.

A conservative estimate of the duration of the TMS effects was 8.5 min (50% of the duration). The behavioral tasks, computer-paced, took 7.5 min. With such a limited time window, we confined our episodic condition to the Imagine Helping task and our control condition to No Helping task from Experiment 1. TMS was applied to the fMRI-defined subject-specific RTPJ in one session and to a control region ~5 cm posterior to the RTPJ in the axial plane that falls outside the functional localizer (see Supplementary Tables 4 and 5). Modeling and experimental work suggests that the spatial resolution of TMS stimulation is 5–10 mm (Kammer *et al*., 2001; Valero-Cabré *et al*., 2007). Thus, the control region falls outside the margins of error, increasing confidence in the anatomical separation between the RTPJ and control stimulation sites. We employed an active stimulation control site and also counterbalancing of order of stimulation site across subjects in order to control for any nonspecific secondary effects of rTMS (e.g. auditory sensations, somatic and tactile stimulation, potential startle effects). We used Brainsight software to create a 3D reconstruction of the fMRI localizer scan for every subject and graphically represented both the RTPJ and the control region (Supplementary Figure 1). These individual images were used to plan, guide and monitor the stimulation in real time using a stereotaxic infrared system, ensuring the every TMS pulse was delivered to the predetermined cortical location (Gugino *et al*., 2001). Whole-brain results for the theory of mind localizer yielded a network of brain regions consistent with Experiment 1 and previous work (Saxe and Wexler, 2005; Scholz *et al*., 2009; Young *et al*., 2010; Schurz *et al*., 2014) with peak activity in right TPJ converging on *x* = 45, *y* = −46, *z* = 13 (Supplementary Table 6). While the broader lateral parietal cortex surrounding the RTPJ is functionally heterogeneous (Mars *et al*., 2013), we addressed the issue of functional heterogeneity by tailoring TMS targets for each individual subject based on peak results from individual subjects’ theory of mind functional localizer using subjects’ own native brain space.

Prior to stimulation, participants read instructions for that study and then completed four practice trials to become familiar with the study design (described in detail below). Participants received feedback on their performance from the experimenter after each trial and were allowed to ask any questions concerning the task. If necessary, practice trials were repeated until participants demonstrated task comprehension. Participants were told to fully engage in the task and to be prepared to provide brief descriptions and ratings about their experience immediately following the experimental trials.

Stimulation then took place for 17 min. Subjects did not receive any specific instructions for stimulation, other than to try to relax their shoulders to minimize neck and shoulder twitches and to alert the experimenters if they experienced any kind of headache. Immediately following stimulation, subjects completed a subset of trials from Experiment 1, consisting of eight stories describing everyday events featuring a person in need of help. After reading a story for 7 s, participants either imagined themselves helping the person in need (Imagine Helping-episodic condition) or considered the writing style and media source of the story of need (No Helping-control condition). Motivated by the restricted time window of offline TMS and informed by piloting work in our lab that revealed a prosocial effect arises as early as the initial construction phase of helping episode (see Supplemental Pilot Experiment), participants had 12 s to generate the imagined event or identify the media source, after which the software moved forward to collect ratings similar to the post-task survey used in Experiment 1. Specifically, Experiment 2 collected ratings of ‘willingness to help’ (how likely would you be to help in this situation?; 1 not at all–7 very willing), ‘perspective-taking’ (how much did you consider the thoughts and feelings of the person in the story?; 1 did not consider–7 strongly considered) and ‘scene imagery’ of the imagined events (the imagined scene in your mind was?; 1 vague–7 clear and coherent), with the addition of an ‘individual closeness’ rating to the person in need (how close do you feel to the person?; see Supplemental Material for new ratings used in Experiment 2; Gino and Galinsky, 2012). Once all eight trials were completed, subjects participated in an additional task to measure ‘spatial distance perception’ (adapted from Parkinson *et al*., 2014). We also collected a ‘general closeness’ rating (in general, how close to other people do you feel?) adapted from related work on self-transcendence (Yaden *et al*., 2017) as exploratory measures. Given the literature on these measures, individual closeness, general closeness, spatial distance measures, we included them out of an abundance of caution to rule out alternative explanations for the role of RTPJ in contributing to the prosocial effect of episodic representation, addressing the possibility that disrupting the RTPJ may influence social closeness in addition to theory of mind. Social closeness was measured because, (i) recent work suggests that a region near the RTPJ may broadly code for psychological distance, including how social close we are to someone else (Parkinson *et al*., 2014), and (ii) the social closeness of targets in need can affect prosocial decisions (e.g., Gino and Galinsky, 2012). That said, we were primarily focused on theory of mind, and thus used a functional localizer and image-guided TMS to target the specific region of the RTPJ implicated in theory of mind.

After completing all tasks and ratings, participants were re-presented with the same stories in a spreadsheet in the same order that they were previously presented, with space for the participants to type in their descriptions of what they imagined or the media source identified during the trials. These short descriptions were used to ensure task compliance (e.g. imagining actually helping the person as opposed to simply imagining the situation of the person in need). After finishing the study, participants were asked to wait an additional 30 min to ensure post-TMS safety, debriefed and thanked for their time. Sixteen stories were randomized between the two sessions for each individual participant. The only difference between the first and second session was the stimulation site. The two sessions were scheduled approximately a week apart. Following the same analytical approach used in Experiment 1, all behavioral analyses were conducted in R v.3.2.4 for Experiment 2.

## Results

### Behavioral effect of episodic processes on willingness to help

**Fig. 4:**
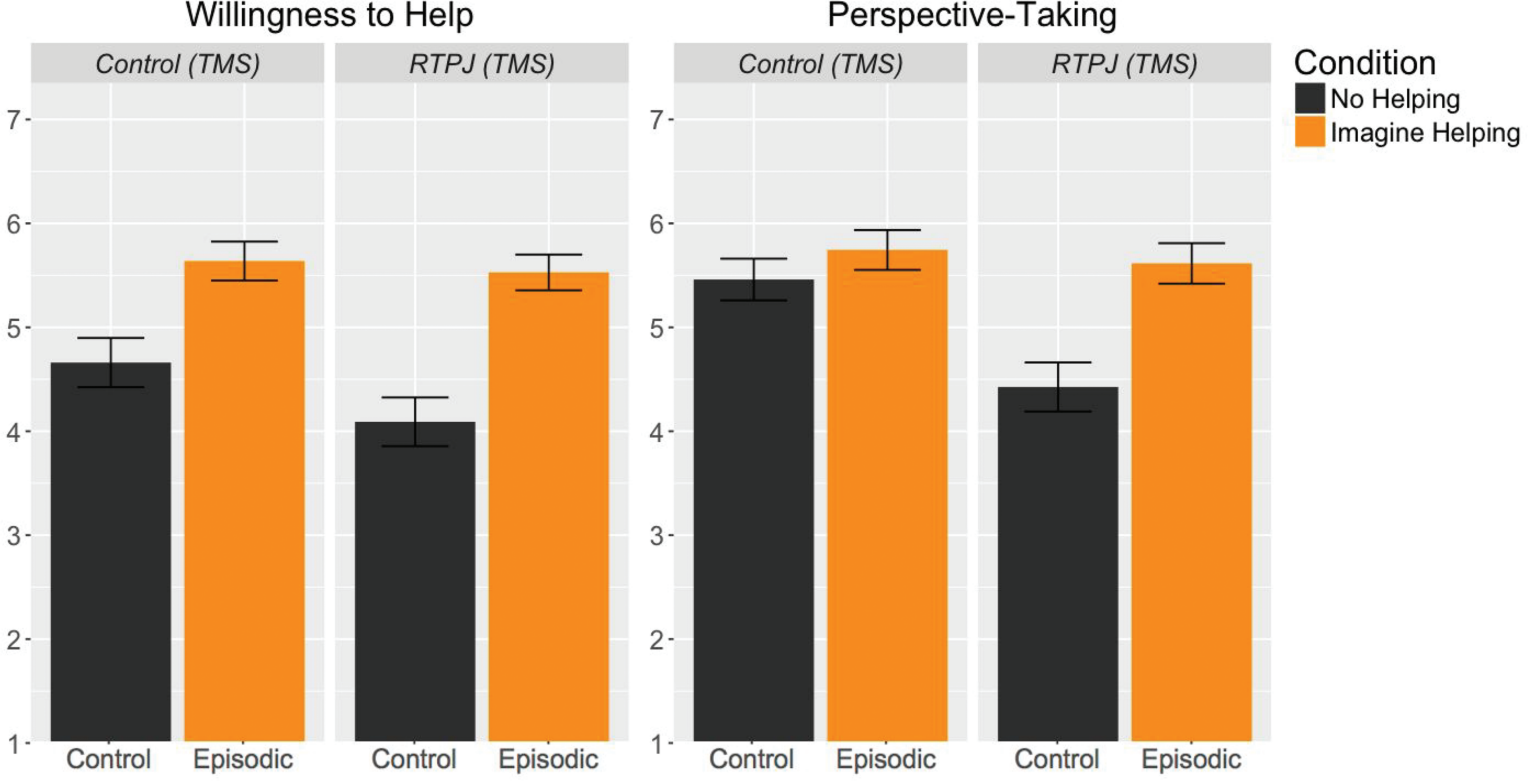
Mean ratings of willingness to help and perspective-taking across control and episodic behavioral conditions, under stimulation to control (TMS) and RTPJ (TMS). We did not observe evidence of an effect stimulating the RTPJ on willingness to help. Stimulating the RTPJ, however, did reduce ratings of perspective-taking in the ‘control’ condition, but not in the ‘episodic’ condition. Error bars indicate standard error of the mean.

To assess the effect of episodic processes on willingness to help, scene imagery and perspective-taking while controlling for variability in mean responses by subject and by scenario, linear mixed models were fit with a fixed effect for condition and random-effects terms for subject and scenario means. Consistent with Experiment 1 and previous work, willingness to help differed significantly across ‘episodic’ and ‘control*’* conditions [*F*(207.23) = 38.75, *P* < 0.001, *d =* 0.77], due to higher willingness to help in the ‘episodic’ (*M* = 5.59) than in the ‘control’ (*M* = 4.38) condition (Figure 4).

Perspective-taking also varied significantly by condition [*F*(181.82) = 14.17, *P* < 0.001, *d =* 0.50] such that perspective-taking was higher for the ‘episodic’ condition (*M* = 5.68) compared to the ‘control’ condition (*M* = 4.92)*.* Individual closeness was also affected by condition [*F*(207.69) = 13.21, *P* < 0.001, *d =* 0.42], such that closeness was higher for the ‘episodic’ condition (*M* = 4.46) compared to the ‘control’ condition (*M* = 3.77).

### TMS results

TMS effects were modeled as a linear mixed model with fixed terms for stimulated region (control *vs* RTPJ stimulation) and for condition, with random-effects slopes for subject- and story-specific region effects. Overall, there was no effect of targeted brain region on willingness to help [*F*(10.60) = 1.77, *P* = 0.21, *d =* 0.22], and no difference in this effect across conditions [*F*(191.51) = 1.50, *P* = 0.20; Figure 4]. Interestingly, although there was no overall effect of targeted region on perspective-taking [*F*(13.12) = 2.46, *P* = 0.14, *d =* 0.38], there was a marginally significant interaction of targeted region with condition [*F*(168.63) = 4.01, *P* = 0.05, Figure 4]. In pairwise comparisons, targeting the RTPJ lowered ratings of perspective taking in the ‘control’ condition [delta *M* = −0.95, *d =* 0.65; *t*(19) = −2.37, *P* = 0.03], but not in the ‘episodic’ condition [delta *M* = −0.19, *d =* 0.11; *t*(22) = −0.45, *P* = 0.65]. We did not observe any effect of TMS on individual closeness (*F*(8.07) = 3.08, *P* = 0.08, *d =* 0.28) or general closeness [*t*(18) = 0.52, *P* = 0.61, *d =* 0.12].

As a further exploration of the potential effects of TMS on spatial distance perception, we adapted a previously used task which assesses participants’ ability to estimate relative distances of objects in photos (Parkinson *et al*., 2014). We did not find any effect of TMS on participants’ judgments of distance, either expressed as subjective distance on a 7-point discrete scale [*t*(18) = 0.19, *P* = 0.85, *d =* 0.08] or as estimated distance in inches [*t*(18) = 0.03, *P* = 0.98, *d =* 0.06].

The results from Experiment 1, revealing that activity in both the MTL subsystem and RTPJ were associated with the prosocial effect of episodic processing, were ambiguous as to whether the MTL subsystem or the RTPJ primarily underlies this effect. The results from Experiment 2 demonstrate that, consistent with previous work, disrupting activity in the RTPJ reduced perspective-taking in control task (Young *et al*., 2010; Santiesteban *et al*., 2012; Ye *et al*., 2015). Interestingly, perspective-taking ratings were resilient to TMS in the episodic task—a finding we will further consider in the general discussion below. Critically, generating a helping episode continued to increase willingness to help to a similar extent when activity in the RTPJ was disrupted with TMS compared to when activity in the RTPJ is intact, suggesting that intact activation in the RTPJ may not be necessary to support the prosocial effect of episodic processing. These findings provide greater insight into the neural basis of this effect, and begin to tease apart the contributions of the RTPJ from the MTL subsystem. While this study did not directly disrupt activity in the MTL subsystem (indeed, to our knowledge, it is currently not possible to directly stimulate the MTL subsystem using TMS), the results of Experiment 2 are consistent with the notion that activity in the MTL subsystem primarily underlies the prosocial effect of episodic processing.

## Discussion

Social neuroscientific research on prosociality has focused on how our judgments about helping people in need are about—and indeed, perhaps driven by—our perceptions of those people’s experiences, intentions, pains and desires. Yet humans also represent information about the environments themselves, and not just the agents who inhabit them. Helping consists of more than representing and reacting to a person in a vacuum: it involves a specific event unfolding in time and place, within which the person is set. Does processing this surrounding information ‘also’ guide our intentions to help?

Here, we present evidence to suggest the answer is yes. The present study finds that neural systems that support imagining future events (episodic simulation) and remembering past events (episodic memory) can also inform willingness to help others. Supporting this link, in Experiment 1 we found that BOLD signal within the MTL subsystem, specifically in the parahippocampus and hippocampus, predicted willingness to help when participants imagined and remembered helping events, suggesting that the neural regions supporting episodic processing can inform prosocial intentions. However, BOLD signal in the RTPJ also showed a similar pattern of activity. In Experiment 2, we found that the effect of episodic helping on willingness to help remained even when activity in the RTPJ was disrupted using TMS.

Interestingly and unexpectedly, the activity in these regions was ‘negatively’ associated with willingness to help when imagining and remembering helping, suggesting that as episodic representations of helping events are more easily constructed they increase our willingness to help in those situations. Providing some support for this account, *post hoc* analysis revealed that perceived task difficulty ratings negatively tracked with willingness to help but only for episodic conditions. This selective pattern is consistent with an ease of episodic construction interpretation (i.e. binding episodic content into an integrated event or scene) as opposed to a general effort interpretation for facilitating prosocial intentions in the present study. While we did not directly manipulate the degree of constructive processing, previous research has shown that activity in the MTL subsystem, including the paraphippocampus and hippocampus, can be modulated and, specifically, reduced through affecting constructive effort via repeated simulations (Gaesser *et al*., 2013; van Mulukom *et al*., 2013; Szpunar *et al*., 2013) or through systematically increasing the amount of information to be integrated into a single event or scene (Summerfield *et al*., 2010). In this last study, increasing the number of elements to be bound together decreased activity in MTL regions along with decreasing perceived integration of elements into a whole and increasing difficulty ratings. Most pertinent to the present findings, activity in the MTL has been shown to be negatively related to perceived probability that an imagined future event would occur (Weiler *et al*., 2010). Considered together, these results are consistent with the idea that, as the helping episode is more easily constructed, as reflected by less activity in the MTL, both the subjective plausibility and willingness to help in that situation increase.

Our neuroimaging results for the MTL subsystem nicely converge with emerging behavioral findings from our lab, revealing that manipulating the spatial representation in which an imagined future episode is located affects willingness to help others (Gaesser *et al*., 2018). In this research, imagined future episodes set in strong spatial contexts increased a willingness to help compared to imagined future episodes set in weak spatial contexts and baseline control conditions. Spatial processing is a critical feature for constructing imagining vivid scenes (see Maguire and Mullally, 2013 for review, but see also Addis and Schacter, 2012 and Andrews-Hanna *et al*., 2010 for related ideas). Specifically, it is thought that the spatial context serves as a platform on which episodic details can be more easily constructed into a coherent and vivid scene (Suddendorf and Corballis, 2007; Andrews-Hanna et al., 2010; Addis and Schacter, 2012). Indeed, episodes based on highly familiar landmarks are brought to mind faster, compared to episodes based on less familiar landmarks (Robin and Moscovitch, 2014; Robin *et al*., 2016).

Our findings add to recent work on social cognition associated with regions within the MTL subsystem. While the MTL subsystem may not inherently support social information processing, there is growing interest in how regions within this subsystem can contribute to social cognition (Croft *et al*., 2010; Tavares *et al*., 2015). However, previous work examining the MTL and social cognition has mainly focused on the contribution of the hippocampus, leaving the contribution of other regions within the subsystem, such as the parahippocampus, underspecified (for reviews see Rubin *et al*., 2014; Schiller *et al*., 2015; Laurita and Spreng, 2017). Additional work has established the role of the parahippocampus in representing spatial contexts and contributing to memory and navigation (Epstein, 2008), but without considering how this role could shape social cognition. The present findings expand our functional understanding of the contribution of these regions to judgments about social interaction.

In the present study, we observed limited evidence that RTPJ, a region associated with theory of mind and the dMPFC subsystem (Saxe and Wexler, 2005; Scholz *et al*., 2009; Young *et al*., 2010; Zaki and Ochsner, 2012; Andrews-Hanna *et al*., 2014), may contribute to the prosocial effect of episodic simulation and memory. In Experiment 1, the activity in RTPJ, similar to activity within the MTL subsystem, was negatively associated with willingness to help for episodic compared to control conditions, thereby making it difficult to isolate the contribution of activity in the MTL subsystem, independent of the similar pattern in the RTPJ. However, in Experiment 2 generating a helping episode continued to increase willingness to help to a similar extent when activity in the RTPJ was disrupted compared to when it was intact, consistent with the notion that activity in the MTL subsystem is differentially engaged compared to the RTPJ and may primarily underlie the prosocial effect of episodic processing. To our surprise, disrupting activity in the RTPJ decreased perspective-taking for the person in need in the control condition, but ‘not’ the episodic condition. Although this effect was not predicted based on previous neurostimulation research (Young *et al*., 2010; Santiesteban *et al*., 2012; Ye *et al*., 2015), one account of this effect is that the episodic condition buffered perspective-taking from being affected by TMS applied to the RTPJ even though perspective-taking was not explicitly elicited in the episodic condition. Stimulating the RTPJ has been recently shown to disrupt functional connectivity to other regions in the theory of mind/mentalizing network (Hill *et al*., 2017). Perhaps imagining helping may have buffered the experience of perspective taking by recruiting compensatory activity in other regions across the theory of mind/mentalizing network. Future research will be needed to test this account. Another interesting possibility to consider is whether episodic representation buffers an effect of TMS on other factors related to the RTPJ that were not directly measured in our study. For example, stimulating the RTPJ has been shown to affect performance on social and temporal discounting tasks in addition to deficits in visual perspective taking (Soutschek *et al*., 2016; see also Hill *et al*., 2017), and it is an open question whether episodic representation would partially buffer these effects, as well.

A limitation of the current set of experiments is that both were conducted before recent efforts to substantially increase power in psychology and neuroscience. We fully support such efforts and recognize that future research building on the present findings would benefit from increased power (increasing the number of trials and subjects). Our designs and samples were selected as they most directly follow the designs of previous behavioral experiments and power analysis of behavioral finings that demonstrate a prosocial effect of episodic processes (Gaesser and Schacter, 2014; Gaesser *et al*., 2015). We note though that the present results are consistent across experiments and converge with previous behavioral findings.

Here, we investigated the impact of episodic simulation and memory on intentions to help. Future work will be needed to investigate whether these judgments will translate into actual prosocial behavior. Prosocial intentions do not always become prosocial actions (FeldmanHall *et al*., 2012). However, we have recently demonstrated that imagining helping can make participants more generous with their money, increasing the amount they donate to individuals in need at an actual financial cost to themselves (Gaesser *et al*., 2018), though the effect of imagining episodes on actual costly prosocial behavior is weaker than the effect on reported willingness to help. A next step will be to meld this behavioral donation paradigm with the current neuroimaging approach to further elucidate how episodic simulation and memory can contribute to prosocial intentions ‘and’ behavior.

Research in social neuroscience and psychology have focused on investigating the contribution of processes underlying our perceptions of people in need and our subsequent emotional reactions to prosociality. Yet, perceiving a need in others does not necessarily motivate helping. Interventions informed by previous research have sought to overcome such prosocial shortcomings by targeting person-centric moral boundaries of group membership and developing a greater sense of empathy. Here we provide behavioral and neural evidence that episodic processes may also facilitate a willingness to help others in need. Our research introduces the intriguing possibility that our capacity for prosociality arises not only from ability to perceive and emotionally connect with others, but from our ability to imagine and remember how to help them.

## Funding

This work was supported by Alfred P. Sloan Foundation grant to L.Y. and the Templeton Science of Prospection Award to B.G., L.Y. and E.K.

## Supplementary Material

scan-18-078-File002_nsz014
