# Supplementary material for: A role for the medial temporal lobe subsystem in guiding prosociality: the effect of episodic processes on willingness to help others
Source: Soc Cogn Affect Neurosci. 2019 Feb 26;14(4):397–410. doi: 10.1093/scan/nsz014 (PMC6523441; doi:10.1093/scan/nsz014)
Supplement: scan-18-078-File002_nsz014 [file scan-18-078-file002_nsz014.docx]

**Supplemental Material**

**Experiment 1**

**Stories of need**

Participants were presented with a series of 40 brief (i.e., one to two sentence stories) depicting everyday events featuring a person in need of help. Stories were adapted from those used in previous work (Gaesser et al., 2016; Gaesser et al., 2015; Gaesser & Schacter, 2014; see Rameson et al., 2012 for related materials) and randomly assigned across conditions:

| While riding the train, this person is harassed by other passengers |
| --- |
| While chopping the vegetables for dinner, this person accidently sliced their thumb. |
| This person's cat recently had kittens and they are causing a mess. |
| This person is locked out of their house. |
| This person left the grocery store and dropped their groceries onto the ground |
| After a series of strong storms, this person came home to find their basement completely flooded. |
| This person was sitting in a park and someone else accidently stepped on and broke their cell phone. |
| This person just finished eatting at a restaurant and is feeling ill. |
| This flower delivery person, who usually uses their own car for deliveries, finds out their car was stolen the day before Valentine’s Day. |
| This person is suffering from dementia and is lost in a mall |
| This person's dog has not returned home in the last 24 hours. |
| Even after attempting to make friends, this person has been sitting alone in the dining hall during meals for the last week. |
| This person’s friend bailed on them after saying they would help them move their furniture into their new apartment. |
| After coming home from work, this person discovered their apartment has a rat problem. |
| This person was stung by a bee; their hand hurts and is swelling up. |
| This new driver wrecked their parents’ car in a snowstorm. |
| In the city, a bike was just stolen from this person, even though it was locked. |
| After returning from the beach, this person realized they're missing the watch their grandfather gave them. |
| This person doesn’t have money for a ticket and is about to miss the last commuter rail of the day home. |
| An earthquake just struck this person’s town, and several buildings have suffered severe damage. |
| This person's neighbor accidentally shot off fireworks into their garage, which may catch on fire. |
| This person's child is a weak swimmer, and is struggling to swim back to shore. |
| The red cross is having a blood drive, and this person is working hard to get people to participate, but everyone is passing them by. |
| Normally this person would deliver meals to the elderly, but they can't make their deliveries tonight. |
| During an argument, this person's housemate began breaking things. |
| The date is approaching for this person’s birthday party, but it is looking like most people won’t be able to make it. |
| This person ate some food that caused them to have a strong allergic reaction. |
| Driving to their wedding, this person’s car broke down on the highway. |
| This person just received a call informing them that their brother has been in a serious car crash. |
| After organizing a fundraising dinner, this person became ill and cannot work in the kitchen. |
| On the way home from school, this person just got into a car accident. |
| This person’s 8-year old child has been running a fever for several days. |
| This person lost their voice the day they are supposed to run a charity auction. |
| This person has just come home to find their apartment has been broken into. |
| This person is quite sick and is having difficulty getting around their house. |
| This person works at a nearby charity, and they not have enough volunteers to help paint children's toys. |
| A friend’s child dropped and broke this person’s treasured family heirloom |
| This person’s roommate spilled their drink all over their expensive theater tickets. |
| During a snowstorm, this person is sitting in a nearby airport when they find out their scheduled flight home for the holidays has been cancelled. |
| This person is baby-sitting a young child that won’t stop crying and they don’t know what to do. |

**Behavioral results**

***The effect of episodic processes on willingness to help***

Pair-wise comparisons: Imagine Helping > Conceptual Helping*,*( *t(33.01)=4.18, p < 0.001*)*;* Imagine Helping > No Helping*,*( *t(33.01)=5.08, p < 0.001*); Remember Helping >Conceptual Helping*,* (*t(41.06)=4.56, p < 0.001*)*;* Remember Helping > No Helping*,*(*t(41.08) = 5.41, p < 0.001*).

***No interaction with gender***

To explore whether gender interacted with an effect of episodic processes on willingness to help and related phenomenology, we re-ran our behavioral analyses with gender entered as a between-subject variable. The effect of *Episodic* (*M* = 5.47) compared to *Control* (*M* = 4.53) conditions on willingness to help did not significantly interact with gender (*F*(1,16) = .22, *p* = 0.645). Pairwise tests showed that the *Episodic* conditions (Imagine Helping and Remember Helping) increased willingness to help to a similar extent for females (*t*(11) = 0.574, *p* = 0.578) and males (*t*(5) = 0.416, *p* = 0.694)*.* Moreover, scene imagery and perspective taking were also similarly matched in the Imagine Helping and Remember Helping conditions for females (SI: *t*(11) = .58, *p* = 0.572; PT: *t*(11) = 1.81, *p* = 0.98) and males (SI: *t*(5) = 1.41, *p* = 0.218; PT: *t*(5) = 1.21, *p* = 0.281. Thus, males showed a similar pattern as females for the *Episodic* conditions on the degree of willingness to help and phenomenology of the helping episode they evoked, consistent with previous behavioral work (Gaesser et al., 2016; Gaesser et al., 2017; Gaesser et al., 2015; Gaesser & Schacter, 2014). Thus, it seems unlikely that our findings in the present study are attributable to an imbalance of gender in the sample and limited to females. Nevertheless, investigating gender differences in the prosocial effect of episodic processes may be an interesting question to examine in the future.

***Episodic behavioral flexibility***

While imagining and remembering helping events had a similar effect on degree of willingness to help and phenomenology of the helping scenario, we did observe evidence of a difference in flexibility. Specifically, miss trials (those on which the subject failed to generate an event) were significantly more common when remembering scenarios (134 hits vs. 39 misses; 29% misses) than when imagining scenarios (177 hits vs. 3 misses; 2% misses; chi-squared = 36.68, *p* < 0.001).

These findings bolsters evidence of a flexible advantage for imagination compared to memory in facilitating prosocial decision-making (Gaesser & Schacter, 2014), expanding the range of prosocial events imagining facilitated compared to remembering. There were some helping scenarios that the participants had not experienced before and therefore could not remember. Contrast this limitation of past events with the fact that participants were able to imagine future helping events on almost every trial, and it underscores a potentially crucial prosocial advantage for episodic simulation over memory: equipping us to help others in novel situations. An exciting direction for future research will be to explore the precise boundary conditions of this flexibility advantage for “filling in” social gaps arising from our inherently restrained past experiences and fostering prosocial decision-making to help in situations we have not personally experienced before.

**fMRI results**

See below for coordinates of medial temporal ROIs derived from (Andrews-Hanna et al., 2010).

| **Supplementary Table 1.** *MNI coordinates for medial temporal ROIs* | | | | |
| --- | --- | --- | --- | --- |
| ROI | *x* | *y* | *z* |  |
| Right hippocampus | 22 | -20 | -26 |  |
| Left hippocampus | -22 | -20 | -26 |  |
| Right parahippocampus | 28 | -40 | -12 |  |
| Left parahippocampus | -28 | -40 | -12 |  |
| Right retrosplenial cortex | -14 | -52 | 8 |  |
| Left retrosplenial cortex | 14 | -52 | 8 |  |

***Whole-brain contrasts***

We examined which regions were commonly recruited when participants engaged in episodic simulation and memory for prosocial events. Whole-brain maps thresholded using FWE correction at *p* < 0.001, clusterwise *p* < 0.05, for the experimental task revealed only a small cluster in posterior cingulate cortex with preferential activation for Episodic (Imagine Helping, Remember Helping) compared to Control conditions (Conceptual Helping, No Helping; Supplementary Table 2).

| **Supplementary Table 2.** *Peak MNI coordinates for whole-brain contrast Episodic > Controls* | | | | | |
| --- | --- | --- | --- | --- | --- |
| Cluster No. | Region | *x* | *y* | *z* | *k* |
| 1 | Posterior cingulate cortex | -9 | -55 | 52 | 4 |

***Whole-brain contrasts, theory of mind localizer task***

We report cluster statistics for the key contrast of inference in the localizer task below:

| **Supplementary Table 3.** *Peak MNI coordinates for whole-brain contrast Belief > Photo* | | | | | |
| --- | --- | --- | --- | --- | --- |
| Cluster No. | Region | *x* | *y* | *z* | *k* |
| 1 | Right temporal pole/right temporoparietal junction | 54 | -49 | 25 | 1176 |
| 2 | Left cerebellum | -24 | -73 | -29 | 36 |
| 3 | Left middle temporal gyrus | -57 | -7 | 20 | 204 |
| 4 | Right frontal orbital gyrus | 48 | 29 | -11 | 39 |
| 5 | Lingual gyrus | -3 | -79 | -2 | 18 |
| 6 | Right inferior frontal gyrus | 54 | 23 | 10 | 43 |
| 7 | Precuneus | 3 | -55 | 22 | 607 |
| 8 | Left temporoparietal junction | -45 | -49 | 22 | 466 |
| 9 | Medial frontal cortex | 3 | 47 | 25 | 96 |
| 10 | Right middle frontal gyrus | 24 | 29 | 43 | 29 |
| 11 | Right superior frontal gyrus | 15 | 29 | 58 | 24 |

***ROI-to-ROI connectivity analysis***

We ran exploratory ROI-to-ROI connectivity analysis using the CONN functional connectivity toolbox (<https://www.nitrc.org/projects/conn)> but did not find any evidence that activity in the MTL was correlating with activity in the RTPJ. Thus, it seems that activity in both MTL and RTPJ is interacting with the episodic conditions to predict willingness to help, but the activity is not co-varying across the MTL and RTPJ.

**Experiment 2**

**Additional ratings:**

Additional ratings used in Experiment 2 include a measure of *individual closeness* rating to the person in need (Gino & Galinsky 2012), *spatial distance perception* (adapted from Parkinson, Liu, & Wheatley, 2014), and *general closeness* adapted from related work on self-transcendence (Yaden, Haidt, Hood, Vago, & Newberg, 2017). Individual closeness, general closeness, spatial distance measures were included in Experiment 2 to explore and address the possibility that disrupting the RTPJ may influence social closeness in addition to theory of mind. This was based on emerging work finding that a region near the RTPJ may code for psychological distance include how social closeness we are to someone else (Parkinson et al., 2014).

***Individual Closeness***


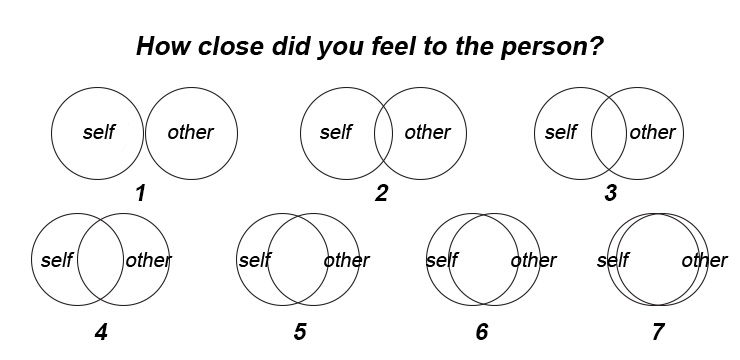


***Spatial Distance Perception***

In this task, you will briefly VIEW a picture of an object in a space, along with a number representing its distance from you in inches.

You will then view two more pictures of the same object in the same space. For each of these new pictures, you will:

1. RESPOND on a 1-7 scale (see below) to indicate **how much further/closer the object is** in this picture relative to Picture 1

1 2 3 4 5 6 7

*much closer*

*neither closer nor further*

*much further*

2. TYPE a number to represent **your estimate of this object's distance from you**

*X inches*

You will have 7 seconds to respond to each picture.

::PICTURE 1 was presented::

(view)


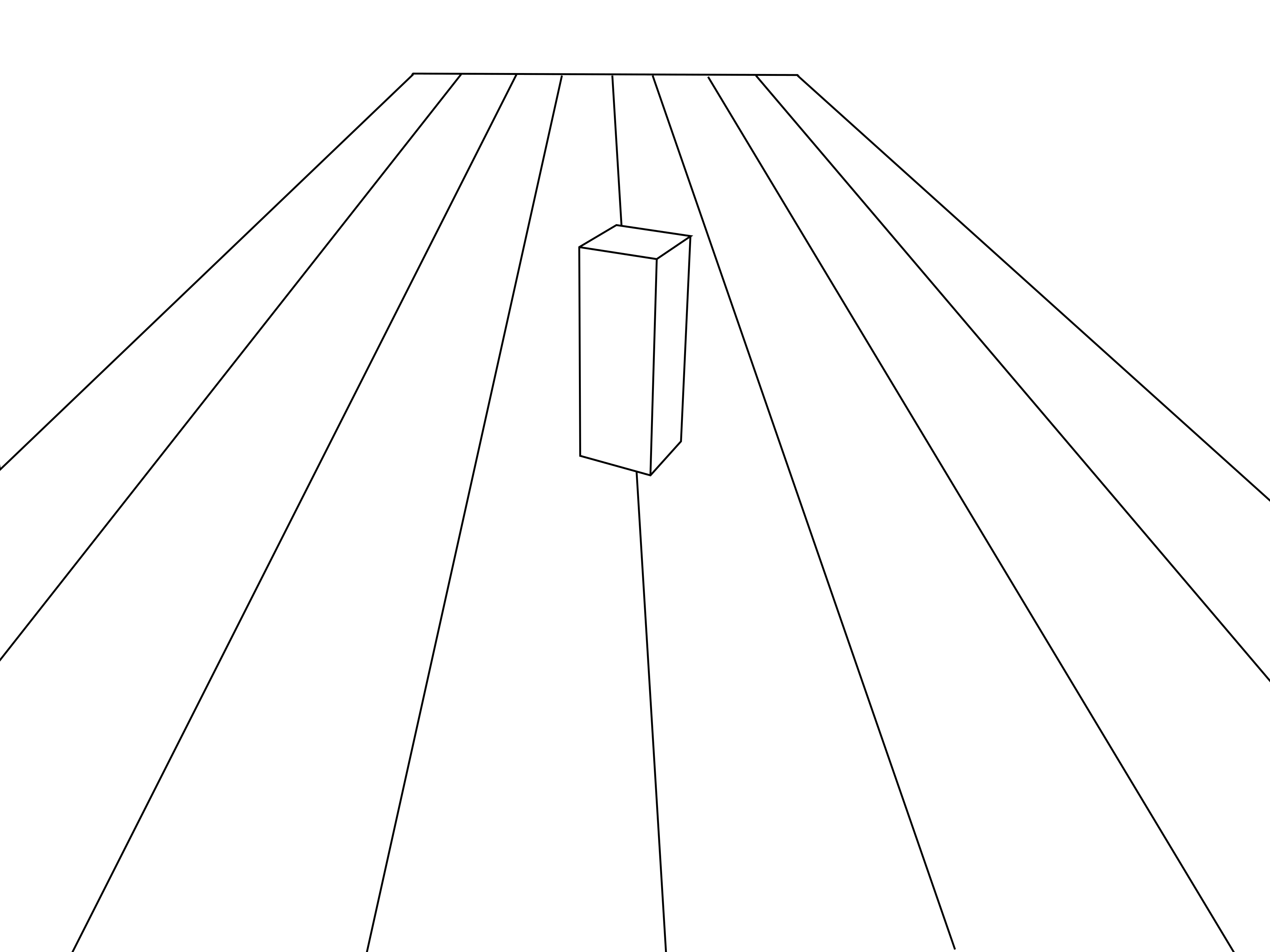


*26 inches*

Distance:

::PICTURE 2 was then presented on a separate screen::

(respond)


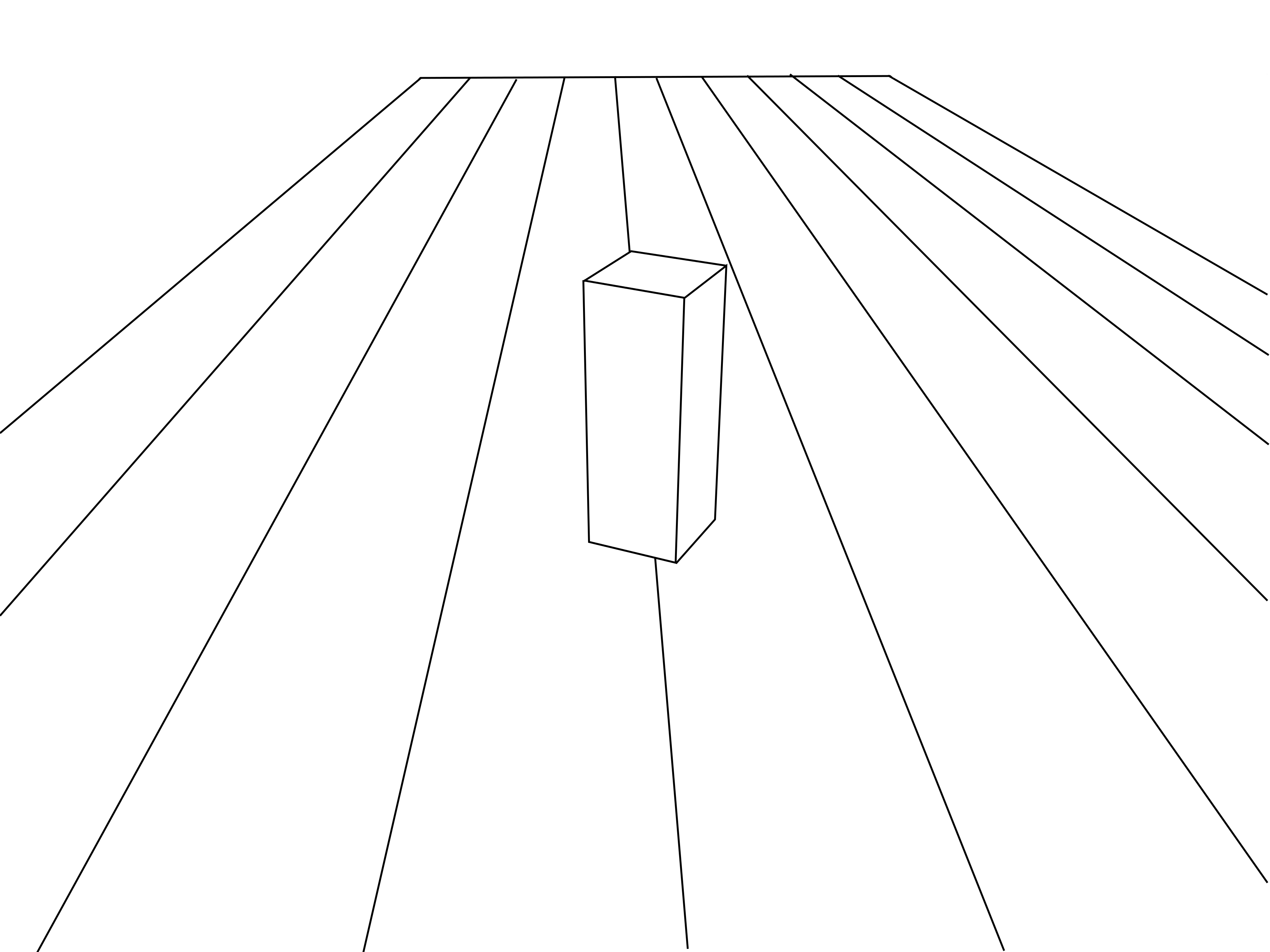


1 2 3 4 5 6 7

*much closer*

*neither closer nor further*

*much further*

*Type here*

Distance:

::PICTURE 3 was then presented on a separate screen::

(respond)


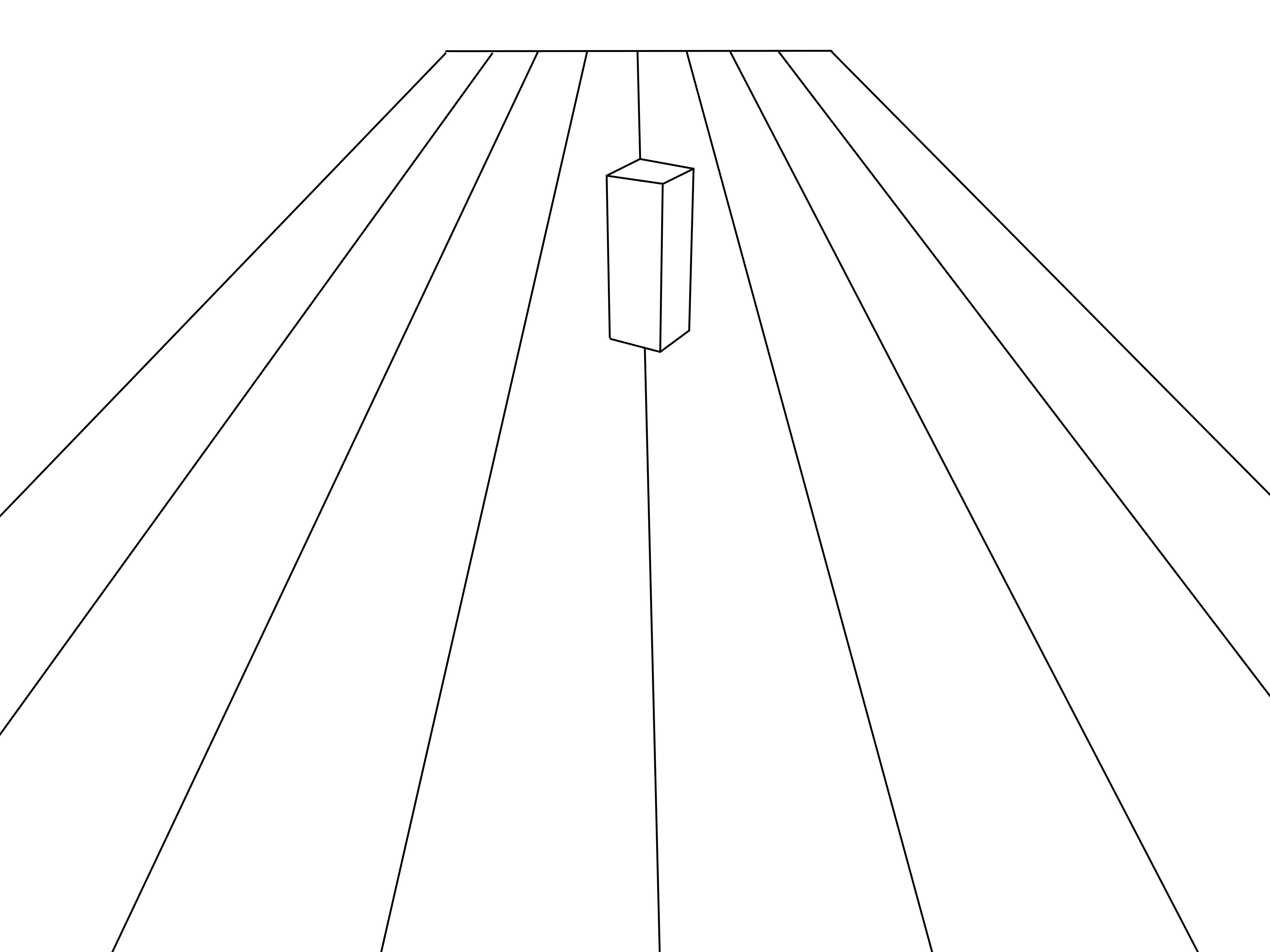


1 2 3 4 5 6 7

*much closer*

*neither closer nor further*

*much further*

*Type here*

Distance:

***General Closeness***

At the very end of the study we collected a single *general closeness* rating.

***In general, how close to other people do you feel?***

**
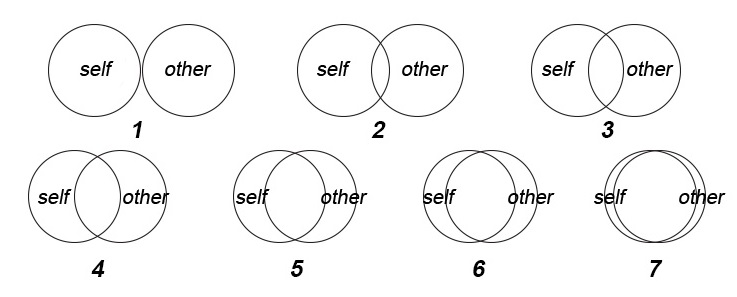
**

**Whole-brain contrasts, theory of mind localizer task**

We examined which regions were commonly recruited when participants engaged in episodic simulation and memory for prosocial events. Whole-brain maps thresholded using FWE correction at *p* < 0.001, clusterwise *p* < 0.05 for for the key contrast of inference in the localizer task below:

| **Supplementary Table 4.** *Peak subject-specific RTPJ coordinates in MNI and native brain space for whole-brain contrast Belief > Photo* | | | | | |
| --- | --- | --- | --- | --- | --- |
| \|  \| RTPJ coordinates (MNI space) \| \| \| RTPJ coordinates (native brain space) \| \| \| \| --- \| --- \| --- \| --- \| --- \| --- \| --- \| \| **Subject** \| **x** \| **y** \| **z** \| **x** \| **y** \| **z** \| \| 1 \| 54 \| -58 \| 19 \| 47 \| -8.3 \| 24 \| \| 2 \| 45 \| -52 \| 31 \| 46 \| -13 \| 38 \| \| 3 \| 57 \| -55 \| 13 \| 50 \| -38 \| 4 \| \| 4 \| 42 \| -58 \| 24 \| 42 \| -44 \| 8 \| \| 5 \| 60 \| -46 \| 34 \| 52 \| -37 \| 20 \| \| 6 \| 42 \| -49 \| 22 \| 46 \| -7 \| 44 \| \| 7 \| 57 \| -46 \| 22 \| 56 \| -16 \| 33 \| \| 8 \| 42 \| -61 \| 16 \| 38 \| -23 \| 33 \| \| 9 \| N/A \| N/A \| N/A \| 54 \| -27 \| 21 \| \| 10 \| 42 \| -52 \| 19 \| 46 \| -24 \| 35 \| \| 11 \| 48 \| -55 \| 19 \| 55 \| -38 \| -7 \| \| 12 \| 48 \| -40 \| 34 \| 59 \| -27 \| 13 \| \| 13 \| 48 \| -49 \| 19 \| 58 \| -17 \| 12 \| \| 14 \| 42 \| -52 \| 19 \| 46 \| -32 \| 29 \| \| 15 \| 45 \| -49 \| 25 \| 51 \| -34 \| 21 \| \| 16 \| 54 \| -49 \| 19 \| 50 \| -30 \| 2 \| \| 17 \| 42 \| -46 \| 37 \| 52 \| -30 \| 2 \| \| 18 \| 42 \| -49 \| 37 \| 52 \| -28 \| 1 \| \| 19 \| 54 \| -43 \| 19 \| 51 \| -29 \| 14 \|   **Supplementary Table 5.** *Peak subject-specific Control coordinates in native space control region in brain native space*   \|  \| Control coordinates (native space) \| \| \| \| --- \| --- \| --- \| --- \| \| **Subject** \| **x** \| **y** \| **z** \| \| 1 \| 42 \| -47 \| 37 \| \| 2 \| 44 \| -52 \| 33 \| \| 3 \| 45 \| -74 \| -5 \| \| 4 \| 52 \| -76 \| -4 \| \| 5 \| 32 \| -71 \| 10 \| \| 6 \| 49 \| -53 \| 33 \| \| 7 \| 58 \| -53 \| 21 \| \| 8 \| 29 \| -65 \| 34 \| \| 9 \| 46 \| -61 \| 6 \| \| 10 \| 44 \| -68 \| 30 \| \| 11 \| 39 \| -78 \| -22 \| \| 12 \| 46 \| -68 \| -7 \| \| 13 \| 57 \| -57 \| 0 \| \| 14 \| 38 \| -66 \| 17 \| \| 15 \| 40 \| -67 \| 11 \| \| 16 \| 46 \| -62 \| -20 \| \| 17 \| 38 \| -74 \| 7 \| \| 18 \| 44 \| -64 \| -6 \| \| 19 \| 53 \| -70 \| 0 \|   **Supplementary Table 6** *Peak MNI coordinates for whole-brain contrast Belief > Photo* | | | | | |
| Cluster No. | Region | *x* | *y* | *z* | *k* |
| 1 | Right temporoparietal junction | 45 | -46 | 13 | 39 |
| 2 | Right middle temporal gyrus | 48 | -16 | -14 | 65 |
| 3 | Left middle temporal gyrus | -51 | -4 | -20 | 22 |
| 4 | Right frontal orbital gyrus | 42 | 32 | -8 | 32 |
| 5 | Right inferior frontal gyrus | 54 | 23 | 13 | 30 |
| 6 | Precuneus | 6 | -52 | 28 | 51 |
| 7 | Left temporoparietal junction | -45 | -52 | 16 | 20 |
| 8 | Medial frontal cortex | 12 | 53 | 25 | 241 |
| 9 | Left dorsal anterior cingulate | -21 | 17 | 40 | 16 |


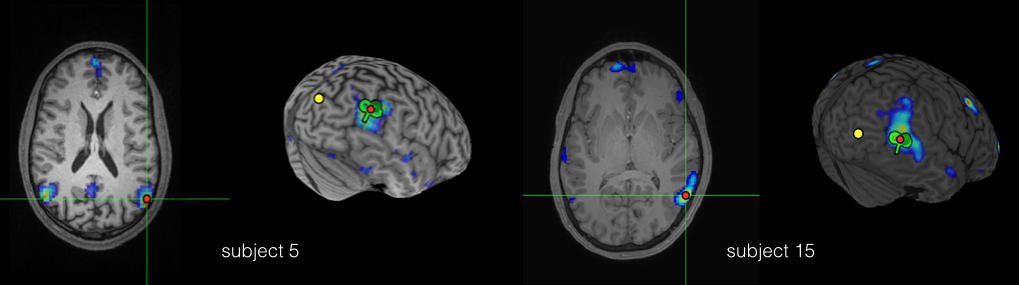


**Supplementary Figure 1.** ToM localizer results and TMS targets in native brain space for two subjects for example-targeting purposes. The subject-specific ToM localizer-defined activity (red-blue) used to identify RTPJ target (green TMS coil) and the control target region that falls outside the ToM localizer-defined activity approximately 5-cm posterior (yellow circle) displayed on structural MRI in the horizontal plane and 3D reconstruction.

**Supplemental Pilot Experiment: Construction vs. Elaboration**

With our TMS equipment and parameters, extensive testing revealed offline TMS could be reliably applied for 17 minutes without overheating the coil. A conservative estimate of the duration of the TMS effects was 8.5 min (50% of the duration) (Young et al., 2010). Thus, we wanted to be able to know if we could elicit a behavioral prosocial effect of episodic simulation in a short time window. Episodic simulation, like episodic memory, is a constructive process that unfolds over time: (i) the initial construction of the imagined future episode when a cue to imagine an event is first presented and disparate details are first integrated and bound together to form an episode that is specific in place and time, (ii) and the subsequent elaboration of the event when the constructed episode is held in mind, and further details are added (Addis, Wong, Schacter, 2007; Addis et al., 2009; Hach, Tippet, & Addis, 2014; Madore, Szpunar, Addis, & Schacter, 2016; see Daselaar, et al., 2008; Ford, Morris, & Kensinger, 2014; Holland, Addis, & Kensinger, 2011 for a parallel distinction applied to remembered events).

In this experiment we sought to test whether the prosocial effect of episodic simulation arises when the imagined helping episode is initially constructed. To do so, we adopted the construction-elaboration paradigm used in previous studies (e.g., Addis et al., 2007, Addis et al., 2009; Hach et al., 2014).

**Methods**

**Participants**

A total of 36 participants were recruited for the study. Participants were students from Boston College and Boston University. All participants provided written informed consent in accordance with the Boston College Institutional Review Board. Participants were run in the lab at Boston College and either received course credit or were paid $15 as compensation. We ran the experiment until we had collected 30 participants (age 18-26 years, *M* = 19.37 years, *SD* = 1.54, 6 males) who provided complete data sets that were then used for analysis.

**Procedure**

Participants consented, completed practice trials, and were presented with the stories of need. After reading a story, participants either briefly imagined themselves helping the person in need without elaborating on the event (Construct Helping condition), imagined themselves helping the person in need in as much detail as possible for one minute (Elaborate Helping condition), or considered the writing style and media source of the story of need (No Helping condition).

For the Construct Helping condition, participants were instructed to imagine an event specific in both location and time in the future that involved their helping out the person in need and pressing the space bar on the computer as soon as they had formed the imagined scene in their mind, at which point the trial ended (see Addis et al., 2007; Addis et al., 2009; Hach et al., 2014, Madore et al., 2016) for similar construction vs. elaboration phase methods). For the Elaborate Helping condition, participants were instructed to imagine an event specific in both location and time in the future that involved their helping out the person in need, and creating an imagined scene in their mind. Participants were prompted to construct and then elaborate on the details of this imagined event for a full 60 seconds, at which point the trial ended. Thus, the Elaborate Helping condition, similar to the Construct Helping condition, involves the initial construction of an imagined helping episode in a spatial context, but also involves generating additional event details. For both conditions involving imagining helping, participants were instructed to imagine plausible events that could occur approximately 1 year from the present to match temporal distance. The No Helping condition from Experiments 1 and 2 served as a neutral control. Participants’ response times for initially constructing an imagined helping event were collected in the Construct Helping condition; ratings of willingness to help for all conditions were collected.

**Results**

**Construct vs. Elaboration Manipulation**

Reaction time results showed that participants were able to generate imagined events in the Construct Helping condition after several seconds (*M* = 7.15, *SE* = .712), consistent with prior work on the initial construction phase of episodic simulations (e.g., Addis et al., 2007, *M* = 7.71; Addis et al., 2009, *M* = 7.50; Hach et al., 2014, *M* = 5.75).

**Willingness to help by condition**

A repeated-measures ANOVA (Elaborate Helping; Construct Helping; No Helping) revealed an effect of condition on willingness to help (*F* (2,29) = 9.03, *p* < .001**,** η_p_^2^ = .237). Paired-samples t-tests revealed participants were significantly more willing to help in the Construct Helping (M = 5.11 SE = .16) condition compared to the No Helping (M = 4.67 SE =.15) condition, *t* (29) = 3.19, *p* = .003. Similarly, participants were also significantly more willing to help in the Elaborate Helping condition (M = 5.30 SE = .17) compared to the No Helping (M = 4.67 SE =.15) condition, *t* (29) = 4.00, *p* < .001. Critically, we did not observe a significant difference in willingness to help between the Elaborate Helping (M = 5.30 SE = .17) and the Construct Helping (M = 5.11 SE = .16) conditions, *t* (29) = 1.15 *p* = .260; (Supplemental Figure 2).


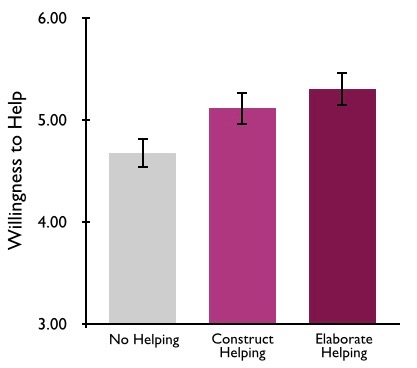


**Supplementary Figure 2.** In a supplemental experiment, initially constructing an imagined helping episode in a spatial context (Construct Helping condition) increased willingness to help above baseline control (No Helping condition) to a similar extent as initially constructing and subsequent elaborating on an imagined helping episode (Elaborate Helping condition). Willingness to help was measured on a 7-point scale, displayed here from 3 to 6 to graphically emphasize statistical similarities and differences across conditions. Error bars indicate standard error of the mean.

**Discussion**

Results showed that both the Construct Helping and Elaborate Helping conditions increased willingness to help to a similar extent, compared to the baseline control condition.

These results suggest the prosocial effect of episodic simulation could be elicited in a short time window, such as would be required to adapt the study parameters for offline TMS.

Beyond prosocial decision-making, our results have implications for studies that have divided the process of imagining future events into construction and elaboration phases (e.g., Addis et al., 2007; Addis et al., 2009; Hach et al., 2014; Madore et al., 2016). These predominately neuroimaging studies have often relied on interpreting patterns of neural activity to make inferences about differences in subjective experience and content across construction and elaboration phases. Methods incorporating cognitive measures across phases and differences on behavioral outcomes may be useful for future work (see Anderson, Peters, & Dewhurst*,* 2015 for related ideas). Here we did not observe differences between construction and elaboration for prosocial decision-making, whether these phases are of consequence to other forms of future oriented decision making remains an open question for researchers to examine.

**Supplemental References**

Addis, D. R., Pan, L., Vu, M. A., Laiser, N., & Schacter, D. L. (2009). Constructive episodic

simulation of the future and the past: Distinct subsystems of a core brain network mediate

imagining and remembering. *Neuropsychologia*, *47*(11), 2222-2238.

Anderson, R. J., Peters, L., & Dewhurst, S. A. (2015). Episodic elaboration: Investigating the

structure of retrieved past events and imagined future events. *Consciousness and*

*Cognition*, *33*, 112-124.

Daselaar, S. M., Rice, H. J., Greenberg, D. L., Cabeza, R., LaBar, K. S., & Rubin, D. C. (2008).

The spatiotemporal dynamics of autobiographical memory: neural correlates of recall,

emotional intensity, and reliving. *Cerebral Cortex*, *18*(1), 217-229.

Ford, J. H., Morris, J. A., & Kensinger, E. A. (2014). Effects of emotion and emotional valence

on the neural correlates of episodic memory search and elaboration. *Journal of cognitive*

*neuroscience*, *26*(4), 825-839.

Hach, S., Tippett, L. J., & Addis, D. R. (2014). Neural changes associated with the generation of

specific past and future events in depression. *Neuropsychologia*, *65*, 41-55.

Holland, A. C., Addis, D. R., & Kensinger, E. A. (2011). The neural correlates of specific versus

general autobiographical memory construction and

elaboration. *Neuropsychologia*, *49*(12), 3164-3177.
